# Supplementary material for: An improved Lagrangian relaxation algorithm based SDN framework for industrial internet hybrid service flow scheduling
Source: Sci Rep. 2022 Mar 9;12:3861. doi: 10.1038/s41598-022-07125-3 (PMC8907310; doi:10.1038/s41598-022-07125-3)
Supplement: Supplementary file 1 — Supplementary Information. [file 41598_2022_7125_MOESM1_ESM.docx]

**An Improved Lagrangian Relaxation Algorithm based**
**SDN Framework for Industrial Internet Hybrid Service**
**Flow Scheduling**

Yan Song^1^, Wenjing Luo^2,*^, Panfeng Xu^3^, Jianwei Wei^4^, Xiangbo Qi^5^,

^1,2,3^Liaoning University, College of Physics, Shenyang, 110000, China
^4^ FieldIoT Co., Ltd; Shenyang, 110000, China
^5^ Shenyang University; Shenyang, 110000, China
^*^lwjwuhua1991@gmail.com

Abstract

The Industrial Internet is the key for Industry 4.0, and network control in the industrial internet usually requires high reliability and low latency. The industrial internet ubiquitously connects all relevant  Internet of things (IoT) sensing and actuating devices, allowing for monitoring and control of multiple industrial systems. Unfortunately, guaranteeing very low end-to-end wait times is particularly challenging because the transmissions must be articulated in time. In the industrial internet, there usually coexist multiple streams. The amount of data for controlling business flows is small, while other business flows (e.g., interactive business flows, sensing business flows) typically transmit large amounts of data across the network. These data flows are mainly processed in traditional switches using a queue-based "store-and-forward" mode for data exchange, consuming much bandwidth and filling up the network buffers. This causes delays in the control flow. In our research, we propose an Software Defined Networking(SDN) framework to reduce such delays and ensure real-time delivery of mixed service flows. The scheduling policy is performed through the northbound Application Programming Interface(API) of the SDN controller so that the dynamic network topology can be satisfied. We use the concept of edge and intermediate switches, where each switch port sends data at a specific time to avoid queuing intermediate switches. We also introduce an improved Langerian relaxation algorithm to select the best path to ensure low latency. Finally, the path rules are deployed to the switches in flow tables through the SDN controller. Our mathematical analysis and simulation evaluation demonstrates that the proposed scheme is more efficient.

Keywords: industrial internet, hybrid service, flow scheduling, SDN framework

1. Introduction

With the emergence of industrial IoT, an increasing number of intelligent devices and infrastructures have formed intelligent systems, as shown in Figure 1. These systems generate time-sensitive data, e.g., system control data, fault monitoring data, etc., transmitted with strict time limits and reliability rules [1]. The original standard Ethernet IEEE802.3 [2] satisfies the bandwidth requirements while maintaining scalability and cost-effectiveness but is not suitable for applications with low latency requirements. In the Industrial Internet, physical processes are controlled by several distributed sensors, actuators, and controllers. Smart Factory is a product of the Industrial Internet, and Cyber-Physical System (CPS) is the key to building SmartFactory.

Cyber-Physical Systems (CPS) depend on a computer network that sends sensor data and actuator instructions to the controller. In general, such CPSs are sensitive systems where network delays and jittering can affect the performance of control of the CPS. When two continuous packets are lost, machines on the production floor may malfunction. For example, Daystar, the third-generation industrial robot developed by Lenovo in 2021, is a CPS system. Daystar industrial robots have been able to work for the manufacture of aircraft, helping workers perform remote painting jobs with precision. First, the robot performs an aspatial scan of the entire workshop for perception. The data is built and rendered in real-time in 3D on an edge-side virtual machine and moved to the designated paint booth through containerized Simultaneous Localization And Mapping (SLAM) technology, navigation, and positioning. The robot’s binocular stereo camera captures the workpiece and surroundings of the operator table in real-time to the edge server. However, this collaborative human-machine interaction is now a bottleneck in the development of industrial robots, as it is necessary to ensure real-time binocular stereoscopic video data return from the robot side, with the camera view following the user’s perspective in real-time, combined with augmented reality technology enabling users to immerse themselves in the dynamic remote environment in real-time. It is also essential to ensure that the robot control algorithm is responsive and robust., this allows the robot in the remote domain to reproduce the action commands from the user with very low latency by taking advantage of the rapidity of transmitting control commands, allowing the robot to operate precisely in the remote environment. But current network speeds are not up to the real-time, low-latency response needed for collaborative robots.

In the field of industrial manufacturing, network interconnection is the foundation. Due to the complexity of industrial communication and automation, improving the delay performance of the network is essential for the future development of industrial communication and automation control. Scheduling algorithms are a crucial part of the network. Efficient scheduling algorithms are closely related to timely and reliable data transmission. The smart factory makes the transmission of mixed data streams a reality. The birth of Software Defined Networking (SDN) provides new ideas for scheduling data flows in multiple data streams. SDN is more suitable for centralized management and control than traditional networks. OpenFlow protocol implements the notion of network programmability, and network state, data flow, and control information can be transmitted in real-time through the standard interface of this protocol; this makes it possible to deploy SDN networks into industrial Internet environments.

Let us enumerate the main contents of this article. 1) this paper introduces the scheduling problem (NP-hard) in the Industrial Internet and introduces an Integer Linear Programming (ILP) model to calculate routing and transmission scheduling to select an optimal path. 2) this paper introduces an improved Lagrangian relaxation algorithm that converges faster than the traditional routing algorithm. The improved algorithm not only ensures the real-time performance of the network but also reduces other cost overheads of the network, such as bandwidth, jitter, and link utilization. In summary, the proposed algorithm overcomes the technical barriers in the field of low network latency, and it is important for the development of network deterministic delay technology.

The remainder of this paper is enumerated as follows. In section 2, this paper present a review of existing studies. In section 3, this paper introduces our network architecture. Section 4 presents our mathematical model of the network architecture. Section 5 describes the improved algorithm for the mathematical model. Section 6，7 discuss the experiment and results.


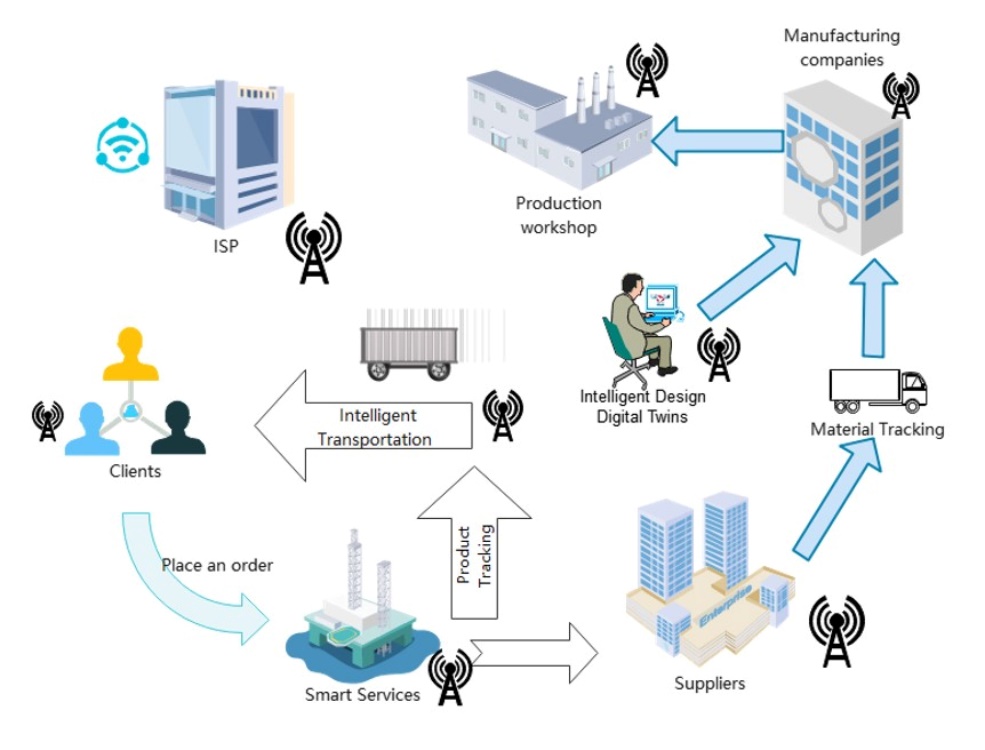


Figure 1. Industry 4.0 Architecture

### Related work

### This paper focuses only on the low-latency problem of scheduling mixed service flows in the industrial Internet. Many scholars have investigated the scheduling of flows. Berclaz et al. proposed the well-known K Shortest Paths (KSP) algorithm [3], which uses the traditional Shortest Path (SP) algorithm to find the shortest path and then finds the subsequent shortest paths based on the initial shortest path. KSP is a static scheduling algorithm; although it performs well in sparse network topologies, the number of traversed paths needs to be increased in dense topologies to achieve optimality. The traditional optimal shortest path algorithm is often unusable due to its high computational effort and is unsuitable for real-time operation. A novel stream scheduling generation model is proposed by Zaiyu Pang et al.[4] The model is designed with two algorithms to suit different application scenarios: the offline algorithm has better schedulability, while the online algorithm consumes less time and has slightly reduced schedulability. Many heuristic search strategies have been proposed to improve the computational efficiency of shortest path search. Misra et al. [5] proposed a polynomial-time heuristic algorithm, but it only considered the maximum throughput under the bandwidth constraint and ignored the delay and packet loss rate. We found a heuristic algorithm based on Particle Swarm Optimization(PSO) to solve the scheduling problem in Xue et al. [6].Nevertheless, the results obtained are not entirely accurate due to the rapid convergence rate, which can lead to an optimal local solution. Even the results do not reflect the actual performance of the PSO. For large-scale scheduling, Pozo et al. [7] proposed a segmentation method that decomposes the scheduling problem into smaller problems that can be solved independently. While maintaining the quality of the schedule reduces the running time of the scheduler and allows scheduling larger networks in a shorter period. The work in [8] relaxes the scheduling rules, divides the Satisfiability Modulo Theories(SMT) problem into multiple Optimization Mode Theory (OMT) problems, and reduces the solver runtime to an acceptable level to eliminate scheduling conflicts. The scheduling is an NP-hard problem. To solve the NP-hard problem, some researchers used the traditional Lagrange relaxation algorithm [9] to transform the relaxation function into a new heuristic function. After analyzing and discussing Lagrange multipliers, we propose multipliers different from the traditional Lagrange relaxation algorithm in this paper.

### To guarantee low latency in the Industrial Internet, the network is traditionally organized by a dedicated Fieldbus network. For this purpose, several real-time Ethernet networks have been proposed, such as ProfiNET [10] and TT-Ethernet[11]. GengLiang et al.[12] used a fieldbus scheduling table to construct an algorithm that minimizes communication jitter.We can assume that the propagation delay, processing delay, and transmission delay in the network are deterministic. The propagation delay, processing delay, and transmission delay in the network are deterministic [13]. Therefore, Niklas Reusch et al.[14] propose a novel and more flexible window-based scheduling algorithm using a time-aware shaper (TAS) that guarantees bounded delays by integrating worst-case delay analysis.These networks architectures limit the non-deterministic queuing delay. For this purpose, Nayak et al. [15] used a Software Defined Network (SDN) architecture and received a series of predefined time-triggered flows. They first proposed an Integer Linear Programming (ILP) formulation to address the problem of combining routing and time-triggered flows. Subsequently, the same authors proposed an increasing flow scheduling and routing algorithm in their paper [16]. This algorithm dynamically adds or removes flows by scheduling them one at a time. The authors propose the concept of a basic period, which is then divided into multiple time slots, each of which is large enough to traverse the entire network with Maximum Transmission Unit (MTU)-sized packets. And this results in too large a time slot being reserved for each stream, resulting in a large amount of wasted bandwidth. The network topology is large enough to introduce time delays with time-triggered flows traversing the entire network. Traditional switches mainly use queue-based "store-and-forward" mode for data exchange processing, where mixed service flows are scheduled in the switch’s "input queue" to different forwarding queues according to priority and then forwarded to the output queue. Inspired by Nayak’s scheduling of time slots for flows to avoid network queuing, we propose an SDN network framework to minimize the possibility of queuing. As shown in Figure 2, the edge switch collects information from end devices and sends it to the SDN controller, which then schedules time slots by the time-sensitivity of different flows. So it can segregate different types of traffic in time and space, thus avoiding queuing problems at the edge switches. It is also inspired by Fan Wang et al.[17] who proposed TracForetime-LSH to perform short-term traffic flow prediction using traffic data detected from sensors.We design a low-latency routing algorithm for messaging between intermediate switch links and also for port data flow prediction to minimize queuing in the intermediate switches. This algorithm effectively eliminates packet loss due to queue overflow while maximizing the number of flows in the network and reducing the end-to-end delay of each flow.

### Network Architecture

### Our network model is based on the software-defined networking (SDN) framework, as shown in Figure 2. SDN is a new network architecture with the separation of the control plane and the data plane. The data plane is responsible for forwarding packets and consists of SDN switches. The control plane accounts for configuring the data plane, for example, calculating routes through related routing algorithms and issuing flow tables to switches. The SDN controller transmits packets to the switches via a southbound interface with OpenFlow(a new protocol in the network) [18]. The SDN controller has the topology and global view of the switch, which helps realize the network control logic (e.g., routing and scheduling in this paper). The data plane of our network architecture consists of switches and end systems (e.g., PLCs, various sensors, and actuators) that support the OpenFlow protocol. The edge SDN switches set priority queues. Based on time sensitivity and latency requirements, the controller divides each flow into specific time slots by the information collected from the flow. The edge switch transmits data to the intermediate switch according to the time slot. The SDN controller calculates the route for the streams from the intermediate switch, selects the best path, and sends down the flow table.

###
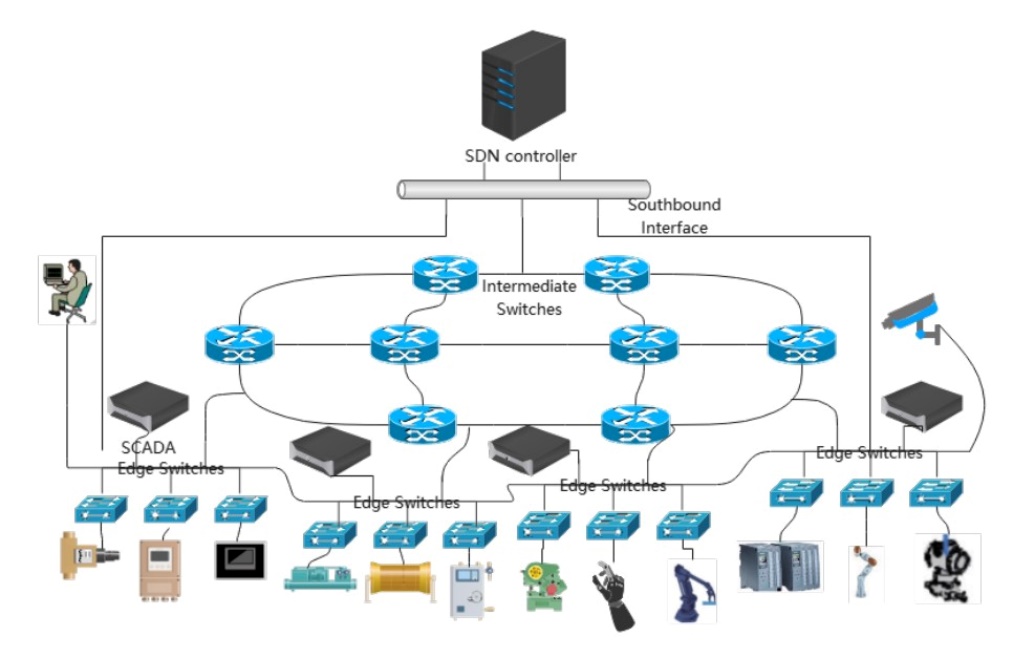


Figure 2. The architecture of Industrial SDN

### Scheduling and Routing Strategy

### The focus of this paper is on routing and scheduling mixed service flows. Our goal is to realize deterministic network behavior and reduce latency in the network and jitter to support real-time communication. Network delays include four kinds of delays, propagation delay, processing delay, transmission delay, and queuing delay. The propagation latency is bounded, which is deterministic and very short, usually in nanosecond units. The processing delay of a commercial Ethernet switch is in the range of less than or equal to microseconds and is approximately constant for a given matched tuple [11]. Therefore, the processing delay can also be regarded as a deterministic delay. For constant traffic, usually, the transmission delay in the network is bounded and definite. When data packets from multiple input ports are transmitted on one output port simultaneously, queuing occurs in the switch. If no two input ports compete for one output port, the source host will only initiate transmission when the traffic passes through a specific path on the network. This situation can be eliminated. Our proposed scheduling algorithm uses the controller’s global information about the network topology and the use of the southbound interface to collect information about various types of flows to define the appropriate transmission scheduling and routing. For example, in the topology shown in Figure 3, different time slots are assigned to each flow Fi, and an optimal path is computed for each flow. In this way, the queuing phenomenon on the bottleneck link will be reduced (from switch S1 toS2) as much as possible. This paper reduces the data transmission delay between end devices, switches, and network controllers by optimizing the scheduling algorithm.

###
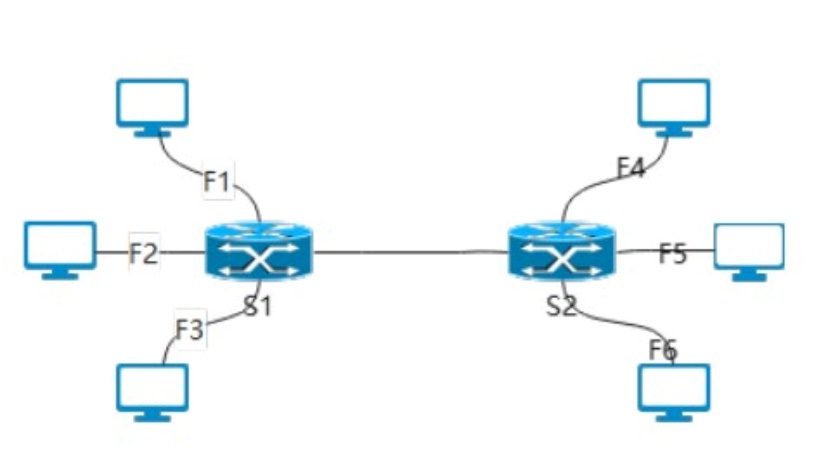


Figure 3. The two-switch-and-six-host topology

### Mathematical Model

### 5.1 Model parameters

We proposed the following mathematical model for the network. In this approach, the scheduler can route flows to any other available path. The inputs are the topology of the network and the start and end positions of a series of flows that need to be scheduled. The variables are the flows that need to be scheduled. The goal of optimization is to maximize the number of flows to be scheduled and to minimize the end-to-end delay of the network.

We propose a directed network topology diagram $G\equiv(N,E)$ , $N$which is a set of nodes, $E\equiv\{(\text{n}_{a},n_{b})|n_{a},n_{b}\in N\}$, which represents a link of the network. denotes a path of a flow$P$,$E\in P$ . In addition, $N\equiv(S\cup C)$, $S$and$C$represent switches and end devices, respectively. A flow is represented as $Fi\equiv(s_{i},d_{i},D_{i})$ , where $s_{i}$,$d_{i}$∈$N$. Here, $s_{i}$is the source of the stream and $d_{i}$ is the destination of the stream. $D_{i}$denotes the delay at which the stream is specified. We define $f(i,m)\in F(i,m)$, where $m\in(n_{a},n_{b})$.

The model requires the following inputs:

-The topology of network,$G\equiv(N,E)$ .

-Characteristics of a series of streams to be scheduled $Fi\equiv(s_{i},d_{i},D_{i})$ .

The decision variable is as shown below:

- We use decision variables to represent the mapping between flows and network links.

$f(i,m)=1$, if the stream$f_{i}$ passes through the link $m$, 0 otherwise.

### Objective function.

We formulate the objective function to maximize the number of schedulable flows while guaranteeing low latency. Therefore, we define the following:

| $Min \sum_{m\in P} \sum_{i\in Fi} f\left( i,m \right)\times d(i,m)$ | (1) |
| --- | --- |

where $d(i,m)$ is the time delay of the stream$f_{i}$ on the link $m$.

| $d(i,m)=[t(n_{a},n_{b})+Q(n_{a,}n_{b})]\times\frac{T(n_{a},n_{b})}{B(n_{a},n_{b})}$, | (2) |
| --- | --- |

where $t(n_{a},n_{b})$ denotes the transmission delay at the top end of the link, $Q(n_{a},n_{b})$ denotes the queuing delay $n_{a}$ on the link $m\in(n_{a},n_{b})$, $T(n_{a},n_{b})$ denotes the average value of accepted packets, and $B(n_{a},n_{b})$denotes the link bandwidth.

### Constraints

- We assume a path i of the flow, which starts from the source host and ends at the destination host. The source host can only have one outbound link without an inbound link, and the destination host is the opposite. For other nodes on the path, the number of inbound links equals the number of outbound links.

| $\sum_{m\in P} f(i,m)-\sum_{m\in P} f(m,i)=\left\{ \begin{aligned} -1,i=di \\ 1,i=si \\ 0,otherwise \end{aligned} \right.$ | (3) |
| --- | --- |

-To avoid conflicts between different streams on the same link, no more than one stream is allowed on any one link.

| $\sum f(i,m)\leq1$ | (4) |
| --- | --- |

- To ensure end-to-end deterministic latency, the total latency of each flow over all links cannot exceed the specified latency.

| $\sum_{m\in P} d(i,m)\times f(i,m)\leq Di$ | (5) |
| --- | --- |

### Many heuristic algorithms can be used when solving integer programming model problems. However, they are complex to implement and depend on the appropriate selection of parameter variance factors. Moreover, complex algorithms consume controller resources. The Lagrangian Relaxation algorithm (LR) [19] can reduce the problem to an unconstrained linear programming problem. Some studies have found that heuristic algorithms about Lagrangian Relaxation techniques are attractive [20]. The basic idea is first to construct a weight and then use the Dijkstra algorithm to find the shortest path. By evaluating a large number of Lagrangian relaxation algorithms for solving delay-constrained problems, we found that the algorithms based on Lagrangian relaxation have low time complexity and a higher probability of achieving feasible or optimal solutions. However, the prime values ​​of Lagrangian multipliers are critical to LR because they may make it difficult for LR to reach the dual optimum or cause a longer time to converge. The computation time of the Lagrangian relaxation method is a serious challenge when the problem size is large. The efficiency of the Lagrangian multiplier update determines the number of subproblems be solved. Traditionally, the Lagrange multipliers are updated using a subgradient-based approach. However, deriving the mathematical formula in the algorithm is very complicated. In the subgradient method, if the step size of the update iteration is not selected properly, it will often increase the number of iterations of the algorithm. The methods for updating the Lagrangian multipliers are finite, and the traditional Lagrangian relaxation algorithm may get caught in locally optimal solutions. BinSahaq et al. [21] proposed a delay-constrained least-cost Quality of Service (QoS) routing approach to finding the optimal Lagrangian multipliers using the Lagrange Relaxation-based Aggregated Cost (LARAC) algorithm. This method operates in polynomial time and produces a theoretical lower bound. Its average path cost is smaller, but it runs slower. They only consider the routing computation task and do not address how to collect information about the network state and available network resources. For this reason, we propose an improved Lagrangian relaxation method that uses a particle swarm algorithm (PSO) to update the Lagrangian multipliers. The reason for using PSO is that it is simple and easy to implement [22] and does not increase the complexity of the original algorithm. And this is faster, more accurate, and easier to converge than the sub-gradient method. Kamboj et al. have also proposed an algorithm to solve the Unit Commitment Problem (UCP) in the power industry [23]. However, unlike their approach, our proposed algorithm updates only one Lagrangian multiplier. Our algorithm is more concise and efficient in solving the scheduling problem.

5.2 Improved Lagrangian relaxation algorithm

We improve the Lagrangian relaxation algorithm with a particle swarm (PSO-LR):

We use the Lagrangian relaxation algorithm to solve the problem described above. The original model is shown below.

| Zlp = min $\sum_{m\in p} \sum_{i\in Fi} f\left( i,m \right)\times d\left( i,m \right)$ | (6) |
| --- | --- |

| s.t. $\sum f(i,m)\leq1$ | (7) |
| --- | --- |

| $\sum_{m\in P} f(i,m)-\sum_{m\in P} f(m,i)=\left\{ \begin{aligned} -1,i=di \\ 1,i=si \\ 0,other \end{aligned} \right.$ | (8) |
| --- | --- |

| $\sum_{m\in P} d(i,m)\times f(i,m)\leq Di$ | (9) |
| --- | --- |

In the LR method, Lagrange multipliers are used to form the penalty terms. After introducing the Lagrangian relaxation operator, the above model can be rewritten as:

$Z_{LR}(\lambda)$= min $\sum_{m\in P} \sum_{i\in Fi} f\left( i,m \right)\times d\left( i,m \right)$-$\lambda\sum_{m\in p} \sum_{i\in Fi} d(i,m)\times f(i,m)-Di$

| = min $\sum_{i,m\in E} (1+\lambda)d(i,m)\times f(i,m)$-$\lambda Di$ | (10) |
| --- | --- |

The Lagrangian dyadic model can be expressed as follows.

| $Z_{LD}=MaxZ_{LR}(\lambda)$ | (11) |
| --- | --- |

To maximize the Lagrangian function, the adjustment of the Lagrangian multiplier must be carefully performed. Most researchers have used the subgradient method to accomplish this task. However, the subgradient method tends to increase the number of iterations. Thus, we use the particle swarm optimization technique to tune the Lagrangian operator (we can also say multipler) in this article, the PSO algorithm is well adapted and simple in practical applications and enhances the performance of the Lagrangian relaxation method. The following is the algorithm flow.

Step 1: Initialize the total number of particles$\lambda_{t}$ (Lagrange operator).

Each particle is randomly generated in the allowed range.

Step 2: Initialize other parameters we needed, such as population size, inertia weights, particle acceleration constants, compression factors, pairwise gaps, etc.

Step 3: Use$Z_{LR}(\lambda)$ to calculate the evaluation value for $\lambda_{t}$each individual in the aggregate.

| $Z_{LR}(\lambda_{t})$=min$\sum_{m\in p} \sum_{i\in Fi} (1+\lambda_{t})d(i,m)_{t}\times f(i,m)_{t}$-$\lambda_{t}Di$ | (12) |
| --- | --- |

Step 4: Compare the value of each individual with its pbest. We denoted the best evaluation value in the pbest as gbest.

Step 5: Alter the membership velocity $n$ for each λ according to the PSO velocity update formula.

Step 6: Update each λ position.

Step 7: If each evaluation value brings better results than the previous pbest, set the latter to pbest. If the pbest is better than gbest, set the value to gbest.

Step 8: We use the idea of ​​dynamic programming to minimize the Lagrangian function for.$\lambda_{t}$.

Step 9: Use to $Z_{LD}$calculate the value of the pairwise function;

| $Z_{LD}=MaxZ_{LR}(\lambda_{t})$ | (13) |
| --- | --- |

Step 10: Use$f(i,j)_{t}$ to calculate the original value:

| J= $\sum_{i,m\in E} d\left( i,m \right)_{t}\times f\left( i,m \right)_{t}$ | (14) |
| --- | --- |

Step 11: The difference between the original problem and the pairwise problem (pairwise gap ε) is used as the termination criterion. The pairwise gap can be calculated by the following equation.

| $\frac{J-Z_{LD}}{J}$ | (15) |
| --- | --- |

Step 12: If the number of iterations reaches the maximum value, or the dual gap is less than the established threshold, go to step 13, if not, then go to step 3.

Step 13: The individual generating the latest information has the shortest delay on each link while scheduling the most streams.

The general flow of the algorithm can be described in Figure 4.


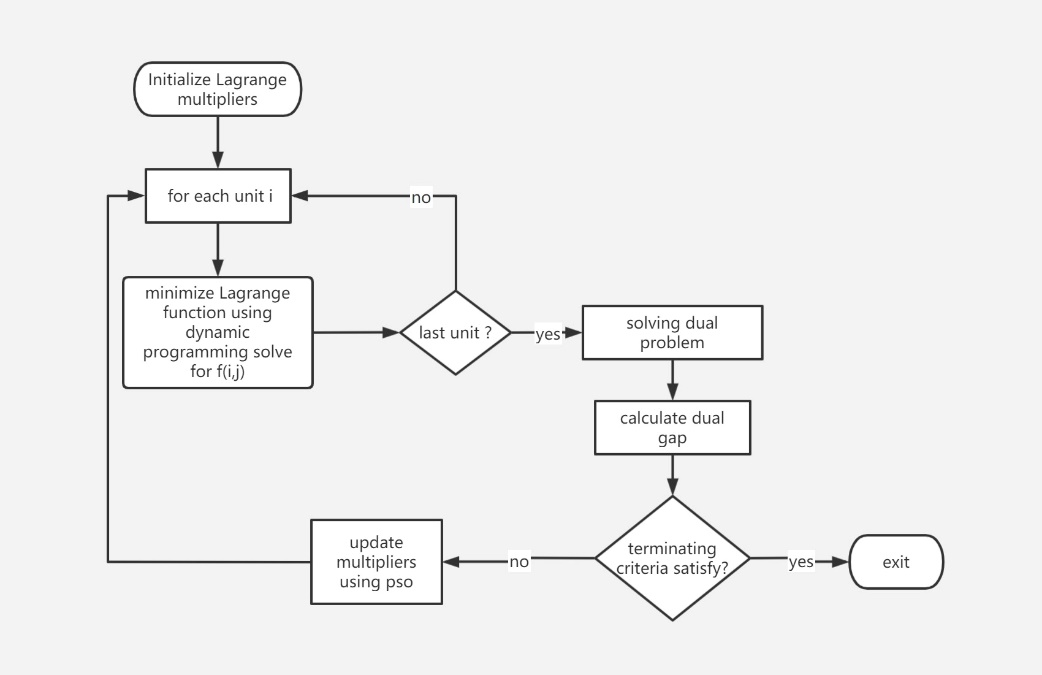


Fig.4 Algorithm flow chart

The algorithm pseudocode is as follows.

| Algorithm PSO- Lagrange Relaxation |  |
| --- | --- |
| input initial population  output the optimal solution   1. Init $\boldsymbol{\lambda}$ 2. While ( iter <= maxcycle , number of generations, or the stopping criterion is not met) 3. For i ∈ Fi 4. for i ← 1 to SIZE(Fi ) 5. do minimize Lagrange function using dynamic programming solve for f(i,m) 6. If (the last unit) then 7. solving dual problem 8. J= $\sum_{\boldsymbol{i,m\in E}} \boldsymbol{d}\boldsymbol{(i,m)}_{\boldsymbol{t}}\boldsymbol{\times f}\boldsymbol{(i,m)}_{\boldsymbol{t}}$ 9. calculate dual gap 10. = 11. If (terminating criteria satisfy ) then 12. update multipliers using pso 13. end if 14. end while 15. end |  |

### The advantage of using particle swarms to update the Lagrangian multiplier is that the PSO algorithm replaces the subgradient method for calculating the Lagrangian multiplier λ, simplifying the theoretical analysis and solution process. It is noteworthy that the presented algorithm has several feasible solutions superior to the original Lagrangian relaxation algorithm. In updating the Lagrangian multiplier λ using PSO, there are many other particles in addition to the optimal one. This provides more alternative models for solving multipath routing problems.

### Experimental settings

### Because of the limitations of the conditions, we configured the switch using Open vSwitch (OVS) and used Ryu as our SDN controller for the convenience of the experiment. We simulated PLCs, signal generators, and actuators with a host computer. We evaluated the algorithm performance using Mininet, an open source platform that uses virtualization on Linux to simulate real-world SDN. Mininet is capable of simulating various types of network components. For instance: computers, switches (layer 2), routers (layer 3), links, and other network elements. For each experiment, we use Mininet to generate topology randomly. The packets from the real-time stream are queued into separate queues in each edge switch. If the flow from a host exceeds the maximum rate set by each queue, we use ingress rules to limit traffic before it enters the switch.

### Netperf is used to create UDP traffic between the source and destination nodes for the stream Fi. Once we have configured the flow rules and queues, the controller assigns a time slot to each flow based on the information collected about the flow. It is sent to the edge switch by downlinking the flow table. The path rules for the streams are sent to the intermediate switch by flow table.

### To evaluate the effectiveness of the mixed flow (e.g., real-time and non-real-time), all nonreal-time streams use separate queues and are served in First In-First Out (FIFO). Since many commercial switches support up to 8 queues per port, in our experiments, we limited the number of real-time streams to 7. We experimented with a single port where each of the seven real-time streams used a separate queue, while the remaining eighth queue was used for non-real-time streams. Our stream rules segregate the two types of streams.

### Scheduling input:

### We take as input the network topology, flow information (including period, route, packet size, deadline), and the maximum time slot window.

### Scheduling output:

### stream sends time per node, arrival time per node in the path the stream travels, time of opening and closing of time slots at each switch egress port, end-to-end delay per stream.

### Our program and algorithms are programmed in the Ubuntu environment using Python, and the program is deployed to the SDN controller. And the data processing results are processed using MATLAB. for convenience; all links are communicated at 1 Gbit/s.

### We evaluate the average end-to-end delay for all flows in each test case, as well as the worst-case end-to-end delay, which is the maximum end-to-end delay in this test case, and the link utilization. We compare the proposed algorithm with the original Lagrangian relaxation algorithm, as well as with Dijkstra's algorithm, which is commonly used for traditional routing.

### Experimental results

To evaluate the performance of our proposed algorithm, we compared it with the Lagrangian relaxation algorithm (LR), Dijkstra's algorithm.

### 7.1 Average delay

The average delay indicates the time needed to transmit data from the source node to the target node. Figure 5 shows the average time delay for different algorithms, and we observe that the average delay increases with the acceleration of data. The average latency varies for different data volumes. The average latency of the three algorithms does not differ much for data volumes up to 100Mb. When the data volume is very large, the average delay of PSO-LR is significantly lower than that of the other two algorithms.

###
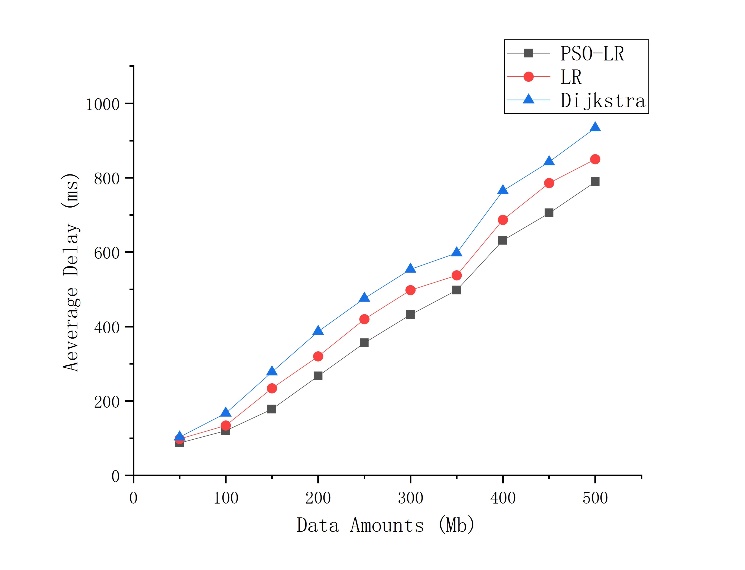


Fig.5 Average delay of PSO-LR, LR, and Dijkstra with different data volumes

### 7.2 Throughput

### Throughput indicates the amount of data successfully received by the destination in the required time. Figure 6 shows the throughput using different algorithms for different amounts of data. This shows that there is a slight variation in throughput at different data load levels. We observe that PSO-LR has the highest throughputs, and thus the most efficient data forwarding path is chosen. The average throughput of Dijkstra, LR, and PSO-LR are 0.15, 0.35, and 0.53 Mb/s, respectively. Namely, PSO-LR throughput obtains the maximum value for different data volumes.

###
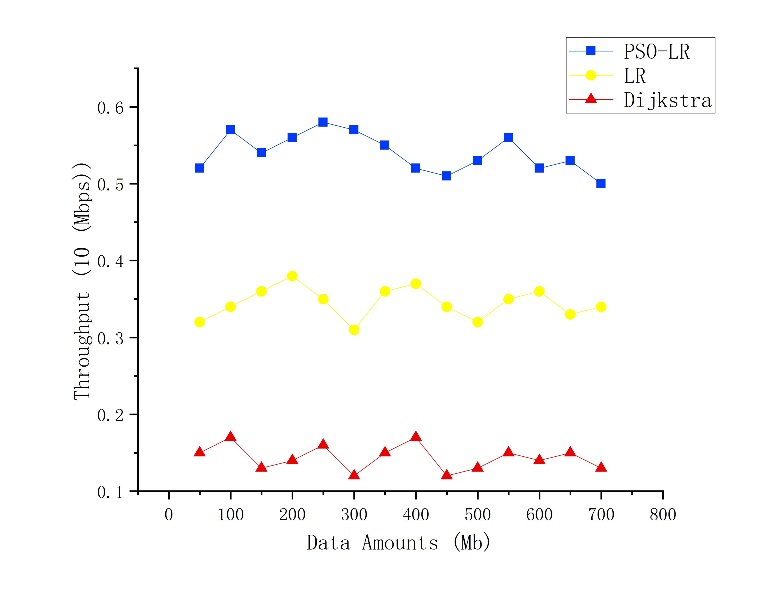


Fig.6 Throughput of data streams

7.3 Latency and loss rate

Figure 7(a) shows the total latency incurred by our proposed algorithm for traffic demands ranging from 10 to 60. The higher the number of service flows, the longer the transmission delay is required, which leads to a longer total delay. When the number of flows exceeds 30, our proposed algorithm generates significantly less delay than the two outside algorithms. Figure 7(b) shows that as the number of flows increases, the packet loss rate also increases. This is because different flows can conflict in the path, which can lead to packet loss. Overall, the packet loss rate can be kept within 0.1%. After the data streams exceed 20, our proposed algorithm significantly reduces the packet loss rate, thus scheduling more streams.

###
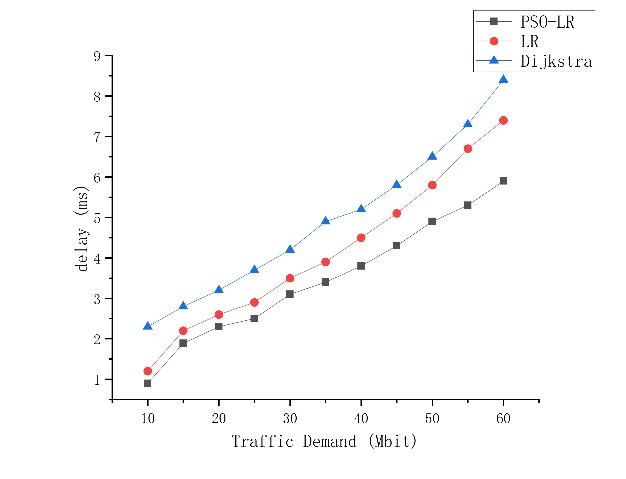

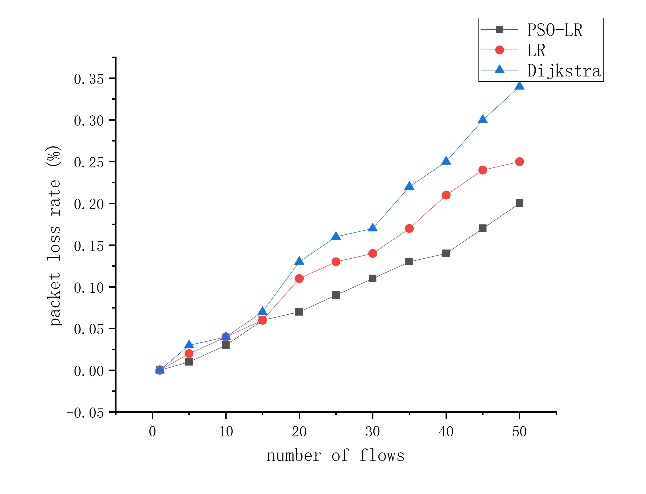


### (a) Total data stream latency (b) Data stream packet loss rate.

### Fig.7 Total delay and packet loss rate with a different number of data streams.

7.4 Total latency

Figure 8 reflects the total latency of real-time streams (RT) and non-real-time streams (NRT) for different numbers of switches. Real-time streams require higher latency and lower end-to-end latency. Using our proposed algorithm, it can be seen that very low latency requirements can be met in medium to large scale networks, even for nonreal-time streams. This is sufficient to meet the latency requirements of the Industrial Internet.

###
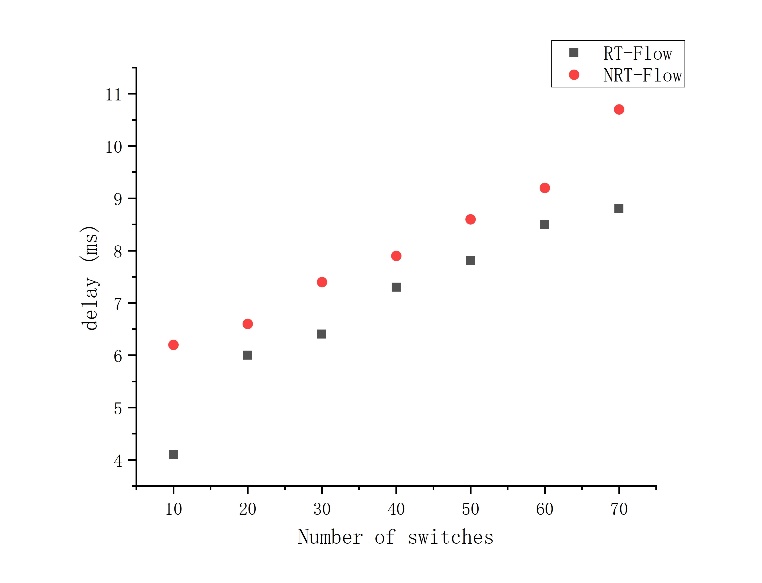


Fig.8 Total Latency of Real-Time Streaming and Nonreal-Time Streaming

### Conclusions and future works

This paper proposes the SDN scheduling framework and the improved Lagrangian relaxation algorithm. The proposed framework can reduce traffic queuing, ensure low delay in traffic transmission, and avoid data retransmission or packet loss due to conflicts in the transmission path of a certain number of scaled flows. Compared with traditional scheduling algorithms, our proposed algorithm can decrease the end-to-end delay by nearly 20% while transmitting more data streams under the condition of consistent network topology. Hybrid flow scheduling in networks is an NP-hard problem that has attracted much attention in the past few years. Here we modify the Lagrangian relaxation algorithm to satisfy the deterministic latency of the transmission network, which embeds the particle swarm optimization algorithm to obtain better performance. This study may have some possible limitations: 1) This study is mainly based on the same network domain. In the real scenario, traffic may be transmitted between different subnets. Scheduling flows between different network domains using SDN is the next topic we will investigate. 2)The experiments in this study are based on computer simulation so that the interference present in the real scenario is ignored. Due to the limited experimental environment, we can extend our work by deploying SDN networks to natural industrial Internet environments. Transport networks can profit from SDN innovations to support and deliver services of high quality.

### References

1. Yager, Ronald R., and Lotfi A. Zadeh, eds. An introduction to fuzzy logic applications in intelligent systems. Vol. 165. Springer Science & Business Media, 2012.
2. 802.3-2015-IEEE Standard for Ethernet. Available online: https:// ieeexplore.ieee.org /document/7428776 (accessed on 4 October 2020).
3. Berclaz J, Fleuret F, Turetken E, et al. Multiple object tracking using k-shortest paths optimization[J]. IEEE transactions on pattern analysis and machine intelligence, 2011, 33(9): 1806-1819.
4. Pang Z, Huang X, Li Z, et al. Flow Scheduling for Conflict-Free Network Updates in Time-Sensitive Software-Defined Networks[J]. IEEE Transactions on Industrial Informatics, 2020, 17(3): 1668-1678.
5. Misra S, Xue G, Yang D. Polynomial-time approximations for multi-path routing with bandwidth and delay constraints[C]//IEEE INFOCOM 2009. IEEE, 2009: 558-566.
6. Xue S, Shi W, Xu X. A heuristic scheduling algorithm based on PSO in the cloud computing environment[J]. International Journal of u-and e-Service, Science and Technology, 2016, 9(1): 349-362.
7. Pozo, F.; Rodriguez-Navas, G.; Hansson, H. Methods for large-scale time-triggered network scheduling.Electronics 2019, 8, 738.
8. Kovásznai G, Biró C, Erdélyi B. Puli–A Problem-Specific OMT solver[C]//Proc. 16th International Workshop on Satisfiability Modulo Theories (SMT 2018). 2018, 371.
9. Sudhakar A V V, Chandram K, Laxmi A J. Lagrange relaxation—Invasive weed optimization for profit based unit commitment[C]//2014 6th IEEE Power India International Conference (PIICON). IEEE, 2014: 1-6.
10. Pigan R, Metter M. Automating with PROFINET: Industrial communication based on Industrial Ethernet[M]. John Wiley & Sons, 2008.
11. Steiner W, Bauer G, Hall B, et al. Time-triggered ethernet[M]//Time-Triggered Communication. CRC Press, 2018: 209-248.
12. Liang G, Li W, Cui Q, et al. An algorithm to build schedule table for schedule-based fieldbus to reduce communication jitter to its minimum[J]. ISA transactions, 2021. ISSN 0019-0578, https://doi.org/10.1016/j.isatra.2021.08.022.
13. Syed A A, Heidemann J S. Time Synchronization for High Latency Acoustic Networks[C]//Infocom. 2006, 6: 1-12.
14. Reusch N, Zhao L, Craciunas S S, et al. Window-based schedule synthesis for industrial IEEE 802.1 Qbv TSN networks[C]//2020 16th IEEE International Conference on Factory Communication Systems (WFCS). IEEE, 2020: 1-4.
15. Nayak, N.G.; Dürr, F.; Rothermel, K. Time-sensitive Software-defined Network (TSSDN) for Real-time Applications. in Proceedings of the 24th International Conference on Real-Time Networks and Systems, New York, NY, USA, 19-21 October 2016; pp. 193 -202
16. Nayak, N.G.; Durr, F.; Rothermel, K. Incremental flow scheduling and routing in time-sensitive software-defined networks. IEEE Trans. Ind. Inform. 2018, 14, 2066-2075
17. Wang F, Zhu M, Wang M, et al. 6G-enabled short-term forecasting for large-scale traffic flow in massive IoT based on time-aware Locality-Sensitive Hashing[J]. IEEE Internet of Things Journal, 2020, 8(7): 5321-5331.
18. McGeer R. A safe, efficient update protocol for OpenFlow networks[C]//Proceedings of the first workshop on Hot topics in software defined networks. 2012: 61-66.
19. Gu F, Askari A, El Ghaoui L. Fenchel lifted networks: A lagrange relaxation of neural network training[C]//International Conference on Artificial Intelligence and Statistics. PMLR, 2020: 3362-3371.
20. Imai A, Nishimura E, Current J. A Lagrangian relaxation-based heuristic for the vehicle routing with full container load[J]. European journal of operational research, 2007, 176(1): 87-105.
21. BinSahaq A, Sheltami T, Mahmoud A, et al. Bootstrapped LARAC algorithm for fast delay‐sensitive QoS provisioning in SDN networks[J]. International Journal of Communication Systems, 2021: e4880.
22. Hsieh S T, Sun T Y, Liu C C, et al. Solving large scale global optimization using improved particle swarm optimizer[C]//2008 IEEE Congress on Evolutionary Computation (IEEE World Congress on Computational Intelligence). IEEE, 2008: 1777-1784.
23. Kamboj V K. A novel hybrid PSO–GWO approach for unit commitment problem[J]. Neural Computing and Applications, 2016, 27(6): 1643-1655.

Competing interests

### The authors declare that they have no known competing financial interests or personal relationships that could have appeared to influence the work reported in this paper.

### Acknowledgements

This work is financially supported by the National Key R&D Program of China, Project Number 2018YFB1700103. Project of Industrial software definition network service platform supporting new network architecture, Industrial Internet innovation and development project 2019, Ministry of industry and information technology of the people's Republic of China.
